# Percutaneous endoscopic treatment for osteoporotic vertebral compression fracture complicated with lumbar spinal stenosis: a case report and literature review

**DOI:** 10.3389/fmed.2025.1623383

**Published:** 2025-11-06

**Authors:** Mingzhi Liu, Kunpeng Su, Wentao Liu, Hongtao Ge, Derong Xu, Chuanli Zhou

**Affiliations:** 1 Department of Spinal Surgery, The Affiliated Hospital of Qingdao University, Qingdao, Shandong, China; 2 Department of Orthopedic Surgery, College of Medicine, Seoul National Universiy, Seoul, Republic of Korea; 3 Department of Orthopedic Surgery, SMG-SNU Boramae Medical Center, Seoul, Republic of Korea

**Keywords:** osteoporotic vertebral compression fracture, lumbar spinal stenosis, percutaneouscement discoplasty, percutaneous kyphoplasty, spine endoscopic

## Abstract

**Background:**

Osteoporotic vertebral compression fractures (OVCFs) and lumbar spinal stenosis are prevalent among the elderly population. The advent of minimally invasive surgical techniques has led to the emergence of percutaneous kyphoplasty (PKP), percutaneous endoscopic unilateral laminotomy and bilateral decompression (Endo-ULBD) as the prevailing treatment modalities for both conditions. However, elderly patients afflicted with a combination of both diseases frequently necessitate staged surgery or intricate surgical trauma with multiple incisions. Moreover, when a concomitant focal spinal deformity is present, conventional strategies frequently yield suboptimal outcomes.

**Case presentation:**

This case presented is that of a 70-year-old female patient with a medical history including hypertension, diabetes, and pacemaker implantation, who has recently developed progressive low back pain and radiating pain to the buttocks and lower extremities bilaterally. Radiological assessments revealed an L3 vertebral compression fracture with sclerotic margins, L2/3 disk-space collapse with a focal kyphotic deformity, and severe L2/3 spinal canal stenosis. A novel approach was employed in light of the patient’s fragile state and the risks associated with surgery, combining Endo-ULBD, PKP, and percutaneous cement discoplasty (PCD). This approach entailed the utilization of the intervertebral space channel as the sole access point and the percutaneous endoscopic incision as the solitary incision. The patient exhibited immediate postoperative pain relief and neurological improvement. Postoperative imaging confirmed resolution of the focal kyphotic deformity. On the second postoperative day, the patient could ambulate independently with lumbar support, and a 14-month follow-up period demonstrated continued clinical stability.

**Conclusion:**

This case demonstrates the feasibility of this surgical approach for older patients with OVCF and LSS. This approach offers a promising alternative to staged or open surgeries, with the potential to minimize operative trauma while ensuring symptom resolution and rapid recovery.

## 1 Background

Lumbar spinal stenosis (LSS) and osteoporotic vertebral compression fracture (OVCF) are prevalent degenerative spinal conditions that predominantly affect the elderly population (1, 2). These conditions are the most common indications for spinal surgery in individuals aged 65 and above (3, 4). LSS typically manifests with neurogenic claudication and lower back pain, while OVCF can lead to spinal instability, kyphotic deformity, and persistent pain, significantly impacting an individual’s mobility and quality of life (5, 6). The potential for complications, including pressure ulcers, thromboembolism, and lung infections, associated with prolonged bed rest results in conservative treatment not being considered for elderly patients (7, 8).

Fusion surgery, which utilizes metal implants to treat both conditions concurrently, carries an elevated risk of surgical failure due to the diminished bone density that is characteristic of osteoporosis (9). Moreover, the prolonged postoperative recovery period and the elevated infection rates associated with fusion surgery render it a less than optimal choice for elderly patients (10, 11).

Percutaneous vertebral kyphoplasty (PKP) and percutaneous endoscopic unilateral laminotomy and bilateral decompression (Endo-ULBD) are two minimally invasive techniques that have gained popularity in recent years (12–14). These techniques have become increasingly preferred due to their less invasive and faster recovery times when compared with open surgery. However, in elderly patients with coexisting OVCF and LSS, staged surgery and complex surgical incisions are often required, which can increase the surgical burden and infection rate (15, 16).

Percutaneous cemented discoplasty (PCD), a procedure first described by P. P. Varga in 2015 for the management of symptomatic segmental instability with an intradiscal vacuum phenomenon (which can cause dynamic foraminal stenosis) and degenerative lumbar scoliosis has been shown to correct lumbar deformity and achieve indirect decompression of the intervertebral foramina (17, 18). At present, it is frequently utilized in conjunction with percutaneous lumbar discectomy, achieving significant results (19). As a pioneering procedure, its capacity to be utilized in conjunction with other interventions for the treatment of lumbar degenerative diseases remains to be fully developed.

Given the patient’s marked frailty and the risks associated with surgery, a new single-stage surgical procedure was employed that utilizes a small incision and a single surgical pathway to perform three procedures: endoscopic decompression surgery to treat spinal stenosis, vertebroplasty to repair the fractured vertebrae, and cemented discoplasty to stabilize spinal mechanics.

## 2 Case presentation

**Figure 1:**
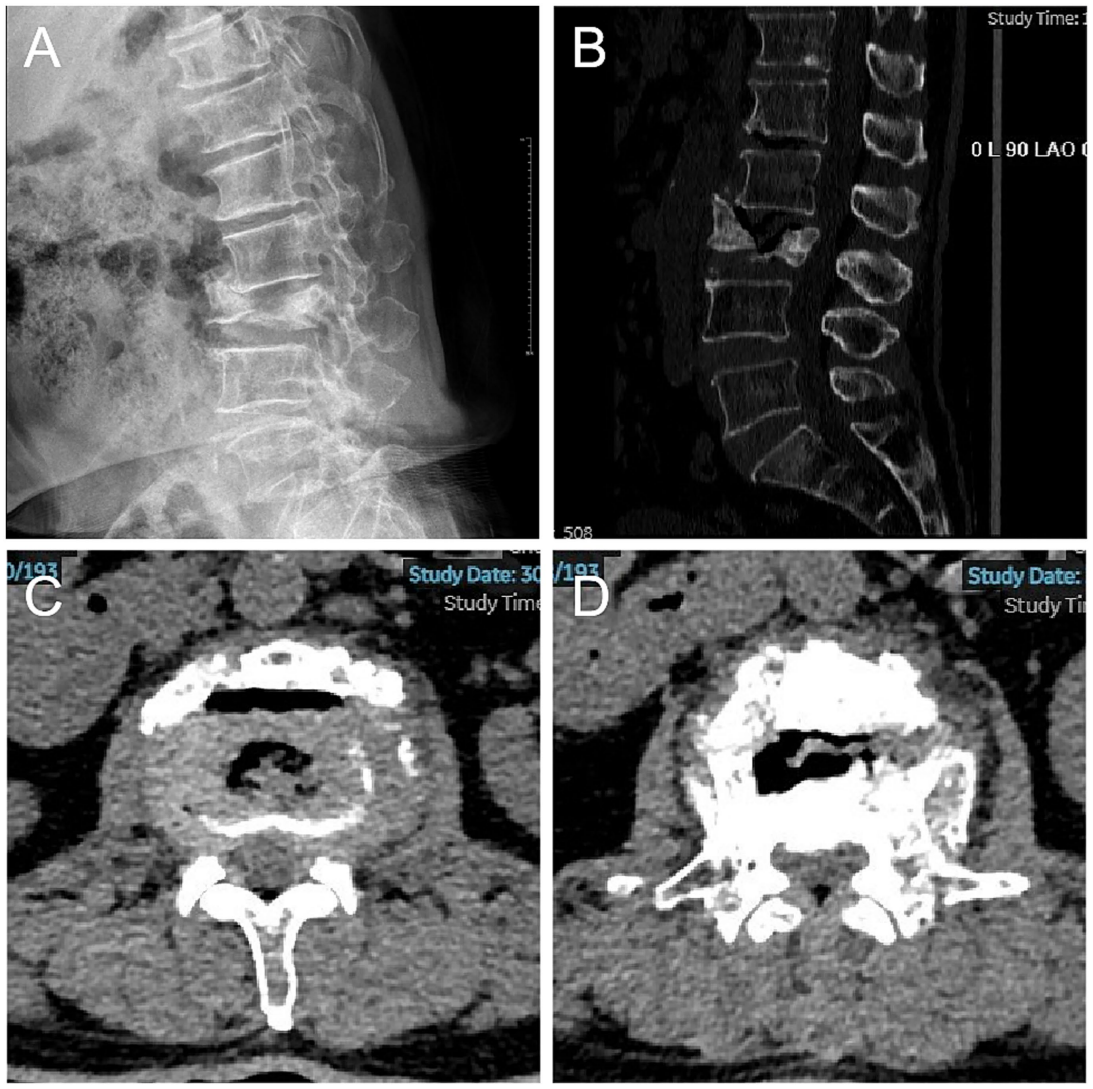
Preoperative radiographs. **(A)** The preoperative lateral lumbar spine radiographs (DR) revealed an L3 vertebral compression fracture with collapse of the L2/3 intervertebral disk space and a focal kyphotic deformity. **(B–D)** Preoperative CT of the lumbar spine further confirmed the L3 vertebral body fracture, accompanied by spinal canal stenosis at the L2/3 level.

A 70-year-old female patient presented with intermittent episodes of lower back pain for the past 2 months, characterized by radiating pain extending bilaterally to the buttocks and lower extremities. The pain radiated anterolaterally down the thighs to the medial side of the feet, with the pain on the left side being more pronounced. The patient and her family denied any inciting trauma, and the symptoms had an insidious onset. The patient had a history of hypertension and diabetes for more than a decade, with satisfactory glycemic control but inconsistent blood pressure management. Four years prior, she had a pacemaker implanted due to bradycardia. Underwent a physical examination 12 months prior to presentation. The DXA scan performed at that time revealed a T-score of −2.8, thus meeting the World Health Organization’s definition for osteoporosis. It is regrettable to note that the patient did not receive effective anti-osteoporosis treatment. On physical examination upon admission, her right lower limb demonstrated grade IV muscle strength, while her left lower limb showed grade III muscle strength. Both sides exhibited positive Hoffmann’s signs. The straight leg raise (SLR) test was positive at 30° on the left and 60° on the right. The visual analog scale (VAS) score and Japanese Orthopedic Association (JOA) score were 8 points and 9 points, respectively. Lumbar spine X-rays and CT scans revealed an L3 vertebral compression fracture with sclerotic margins, L2/3 disk-space collapse with a focal kyphotic deformity, and L2/3 spinal canal stenosis (Figure 1). An opportunistic CT-based Hounsfield unit (HU) assessment of trabecular bone at L1 (mid-vertebral cancellous ROI, excluding the cortical shell and endplates) yielded 80 ± 36 HU, below the ~110-HU threshold associated with osteoporosis and vertebral fragility fractures (20, 21). Due to the presence of a cardiac pacemaker, an MRI examination could not be conducted. Preoperative diagnosis: L3 osteoporotic vertebral fracture combined with L2/3 spinal stenosis.

Prior to surgery, a C-arm fluoroscopy guided transforaminal selective nerve root block was performed at the L2/3 level for diagnostic localization. The patient reported significant temporary relief following the block, confirming the L2/3 segment as the responsible level. Given the coexisting L3 compression fracture and the patient’s poor physical status, a one-stage minimally invasive procedure combining decompression, vertebral augmentation, and discoplasty was planned under general anesthesia.

**Figure 2:**
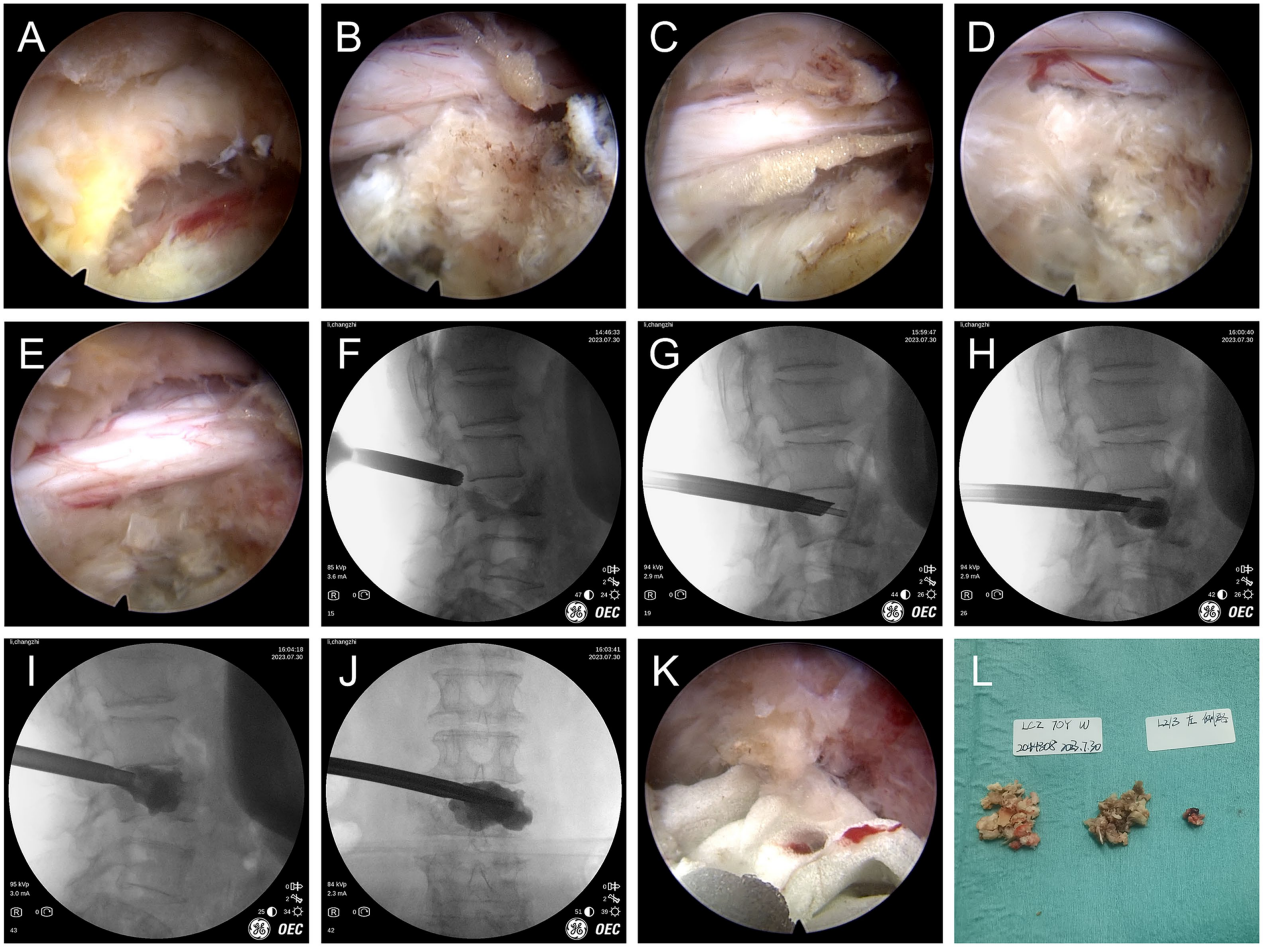
Surgical procedure. **(A)** Exposure of the attachment of the ligamentum flavum. **(B)** Bony decompression of the medial superior articular process with a Kerrison rongeur to decompress the traversing nerve root. **(C)** Probing of the lateral aspect of the nerve with a dissector to confirm adequate decompression. **(D)** Ipsilateral discectomy for decompression. **(E)** The nerve root is freely mobile and the dural sac is well expanded. **(F-J)** Through the working cannula, via the channel used for decompression, the bone cement injector is guided into the vertebral body, and the bone cement is continuously injected, accompanied by biplanar fluoroscopy to avoid leakage of the bone cement, until the bone cement fills the vertebral body and the intervertebral disk. **(K)** Solidified cement under endoscopic visualization. **(L)** Surgical specimen.

The patient was positioned prone with padding under the chest and iliac crests to reduce abdominal pressure. Under C-arm fluoroscopic guidance, the left L2/3 interlaminar space was identified as the entry point. A 7 mm skin incision was made, and a working channel was established using a sequential dilator system. Endo-ULBD was first performed. Under direct visualization, part of the left L2 inferior facet joint, L2 inferior vertebral plate, and L3 superior vertebral plate were removed with a ring saw, thereby exposing the ligamentum flavum attachment points. Partial bone removal of the spinous process root of L2-3 and decompression along the ventral aspect of the contralateral lamina were performed. After removal of the ligamentum flavum, the dural sac and bilateral nerve roots were exposed. The protruded nucleus pulposus was excised to achieve thorough decompression. Exploration revealed relaxed nerve roots and satisfactory expansion of the dural sac (Figure 2).

Then, the endoscope was withdrawn while the working cannula was maintained in position. A balloon catheter was introduced through the working cannula and advanced along the percutaneous endoscopic decompression corridor to the L2/3 intervertebral disk, then directed across the disk space (disk–vertebra route) into the L3 vertebral body. The balloon dilatation compacts the cancellous mass within the vertebral body to form a cavity. Bone cement (polymethylmethacrylate, PMMA) was then prepared and injected into the L3 vertebral body through the same channel under biplanar fluoro, completing PKP. To correct the focal kyphotic deformity in order to relieve foraminal compression, additional bone cement was injected into the L2/3 disk using the same channel, completing PCD (Figure 2). During both kyphoplasty and discoplasty, cement was delivered in small incremental aliquots of approximately 0.2–0.3 mL under continuous biplanar fluoroscopic monitoring, and injection was stopped once the planned filling pattern was achieved.

**Figure 3:**
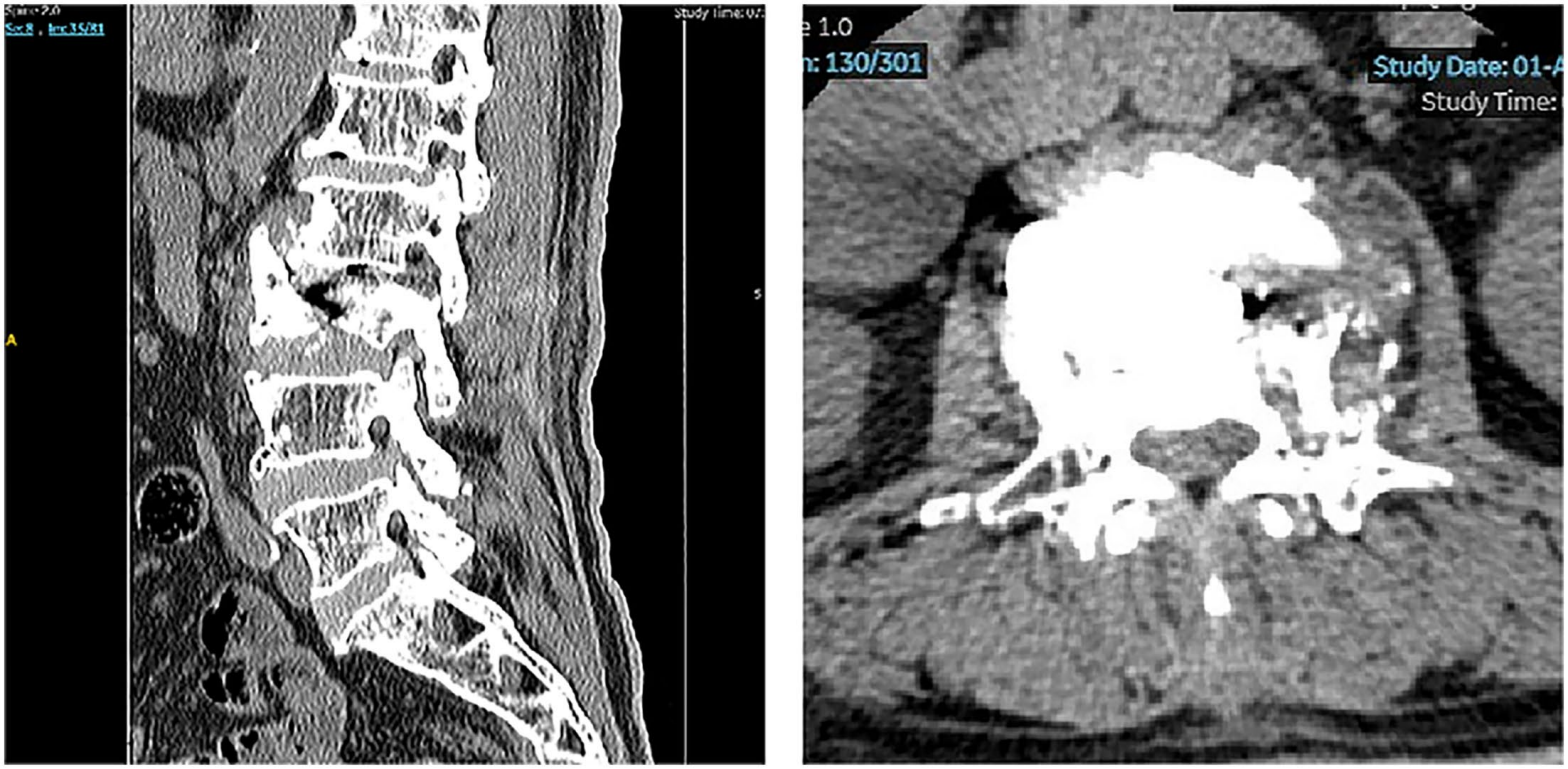
Postoperative lumbar radiographs and CT. Cement is satisfactorily placed within the L3 vertebral body and the L2/3 intervertebral disk. Postoperative imaging demonstrates resolution of the focal kyphotic deformity, restoration of normal spinal alignment, and complete decompression of the L2/3 neural foramen and lateral recess.

The position and distribution of the cement were meticulously monitored with biplanar fluoroscopy to prevent leakage. The incision was closed with a single stitch, and no drainage was required. The total operative time was 105 min, and the estimated blood loss was less than 10 mL. After recovering from anesthesia, the patient experienced significant relief from lumbar and bilateral lower limb pain, with muscle strength returning to normal levels. On the second day postsurgery, the patient was able to ambulate independently with lumbar support. On postoperative day 4, imaging confirmed resolution of the focal kyphotic deformity. The patient was subsequently discharged with stable blood glucose and blood pressure (Figure 3). A one-month follow-up revealed that the patient had made a full recovery, as indicated by the absence of any abnormal symptoms or signs. The VAS score was 1, and the JOA score was 23. Fourteen months following the surgical intervention, the subsequent evaluations revealed no significant variations from the preceding assessments, with no complications observed.

This case exemplifies the efficacious execution of a single-incision, single-channel strategy that integrates three minimally invasive spinal procedures, thereby offering an effective alternative for elderly patients with complex spinal pathology who are not eligible for open or staged surgery.

## 3 Discussion and conclusion

OVCF complicated by LSS are prevalent among the aging population, often resulting in persistent pain, progressive neurological symptoms, and functional decline. The optimal surgical method for patients with a combination of the two diseases has yet to be determined (7, 22).

In our case, we present a novel surgical strategy that integrates Endo-ULBD, PKP, and PCD through a single interlaminar working channel and incision. This one-stage, one-channel surgical plan not only reduced operative trauma but also facilitated simultaneous decompression and stabilization without the necessity of posterior instrumentation or staged intervention. In comparison with conventional open surgery and staged minimally invasive approaches, our technique has been shown to markedly reduce surgical complexity and potential cumulative risk.

A review of the extant literature reveals a range of surgical strategies that have been explored for the management of concurrent OVCF and LSS (Table 1). For instance, Miller et al. (22) and Bonaldi and Cianfoni (23) reported in 2008 and 2012, respectively, on the use of PKP combined with percutaneous interspinous spacer (IS) placement to treat weak elderly patients with OVCF and accompanying neurological deficits. A study by Masaaki Machino et al. (24) compared posterior lumbar interbody fusion (PLIF) with combined anterior and posterior surgery (PACS), reporting good outcomes at the cost of higher surgical complexity. Concurrently, Fukuda et al. (7) demonstrated the efficacy of OLIF in conjunction with posterior spinal fusion (PSF), though they also noted complications, including adjacent segment fractures. Conversely, our approach offers immediate and direct decompression and stabilization through a solitary channel, exhibiting minimal invasiveness and the absence of hardware implantation. This merits particular mention when considering fragile, high-risk elderly patients.

**Table 1:** Treatment modalities for elderly patients with combined LSS and OVCF in the literature.

Literatures	Number of cases	Surgical methods	Follow-up period (months)	Clinical outcome
Miller Jdan et al. (22)	2	PKP combined with percutaneous IS placement	5–18	All underwent secondary PKP surgery.
Bonaldi et al. (23)	4	PKP combined with percutaneous IS placement	12–36	The pain and neurological symptoms disappeared, and no complications were observed.
Chen et al. (32)	87-year-old man	PKP	12	The pain and neurological symptoms disappeared, and no complications were observed.
Lin et al. (16)	15	Staged surgery: One-stage: PKP/PVP Second-stage: Transforaminal full-endoscopic lumbar foraminoplasty and/or discectomy	12	The pain and neurological symptoms disappeared, and no complications were observed.
Mehta et al. (33)	40	posterior approach for decompression, stabilization, and anterior vertebral cleft/body grafting	24	Neurological function: Fully recovered (37 cases); Deteriorated (1 case); Unchanged (2 cases); Unable to walk (2 cases). Complications: Superficial infection (2 cases); Urinary tract infection (2 cases); Pneumonia (1 case); Implant-related complications (3 cases); Adjacent segment fracture (2 cases)
Ishimoto et al. (15)	80-year-old woman	Endoscopic lateral approach to the intervertebral foraminal decompression	36	The pain and neurological symptoms disappeared, and no complications were observed.
Machino et al. (24)	PLIF: 15 PACS: 21	Posterior lumbar interbody fusion (PILF) OR Combined anterior and posterior surgery: anterior decompression and interbody fusion, posterior screw fixation (PACS)	40	Pain and neurological symptoms disappeared, and some patients developed complications. Delirium (1); Superficial infection (1); Secondary surgery (1); Screw loosening (1); Adjacent segment fracture (1) There was no significant difference in complications between the two surgical groups.
Tani et al. (34)	5	Combined application of three minimally invasive surgical techniques: bone cement-enhanced percutaneous pedicle screw placement, lateral lumbar interbody fusion of adjacent vertebrae, and adjacent segment PPS rod implantation	27–48	The pain and neurological symptoms disappeared, and no complications were observed.
Fukuda et al. (7)	20	OLIF and PSF surgery are performed on different vertebral segments according to different types of vertebral collapse.	21–57	Pain and neurological symptoms disappeared, and some patients developed complications during follow-up. Adjacent segment fracture (3)

From a technical perspective, the incorporation of PCD as a component of the intervention is of particular value. The injection of cement into the collapsed intervertebral disk provides anterior column support and may indirectly decompress neural foramina, offering biomechanical benefits while avoiding pedicle screw placement (25, 26). In light of the inherent limitations of osteoporotic bone, this methodological approach has the potential to reduce the incidence of hardware-related complications that have been observed in fusion surgeries (27, 28).

**Figure 4:**
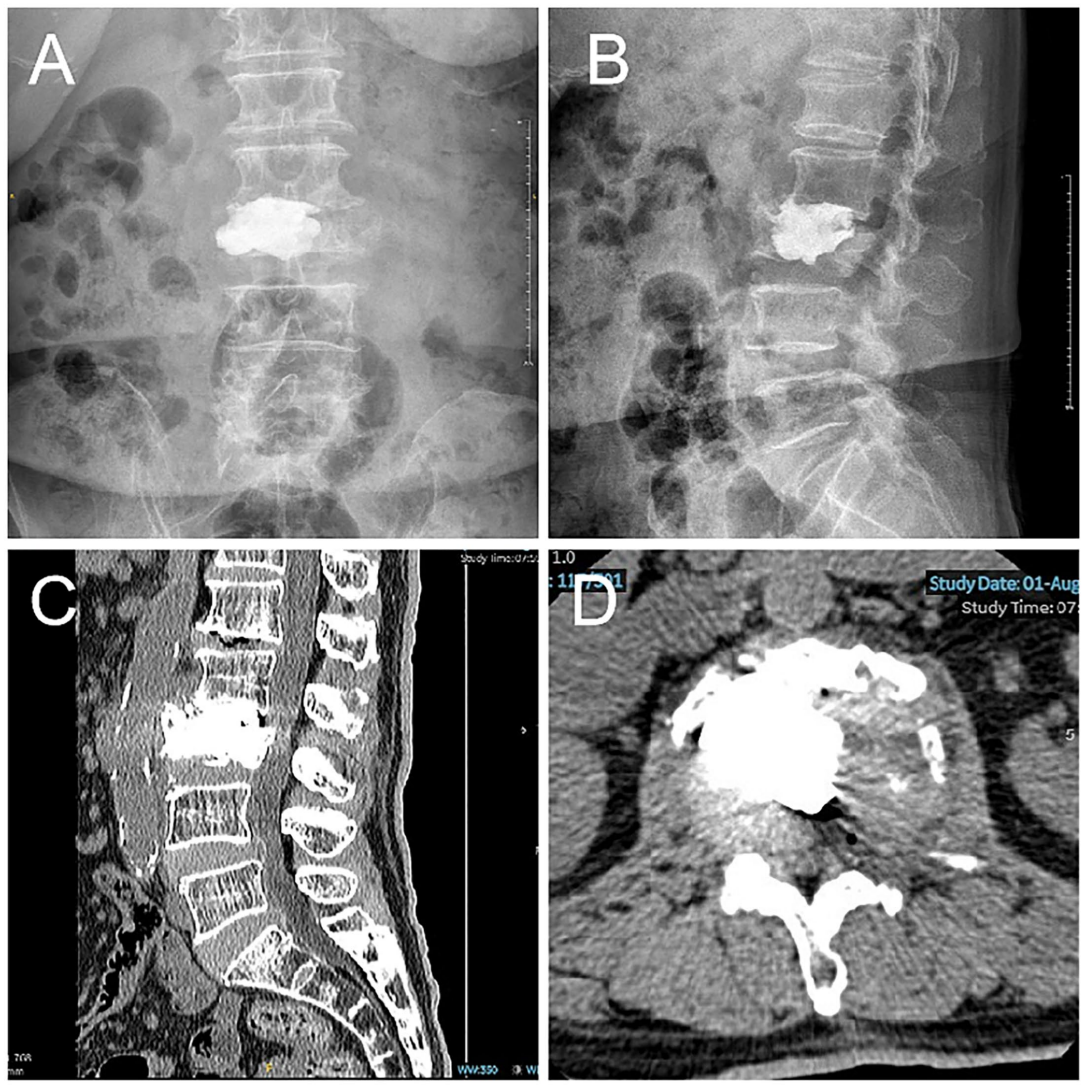
Postoperative lumbar CT scan indicates partial absence of intervertebral disk bone cement. (**A–B**) Lumbar Spine Digital X-Ray Radiography. (**C–D**) Lumbar spine CT.

However, it is imperative to acknowledge the limitations of this approach. Firstly, it should be noted that this report describes a single case, and thus the generalizability of the technique remains uncertain. Although short-term clinical and radiographic outcomes were favorable, long-term follow-up is necessary to assess the durability of vertebral height restoration and risk of adjacent segment degeneration or recurrent collapse. Furthermore, the postoperative follow-up CT revealed the presence of partial absence of bone cement in the intervertebral disk due to inadequate local filling (29) (Figure 4). This may result in complications or prognostic risks that necessitate further follow-up and sample studies.

Patient selection is also a critical consideration. The described procedure may be most appropriate for elderly individuals with coexisting single-level LSS and OVCF, especially when accompanied by intervertebral disk-space collapse, and for those with significant surgical risk due to advanced age or comorbidities. It may not be suitable for patients with multi-segmental instability, severe kyphosis, or those requiring long-segment fixation (30, 31).

In conclusion, our case demonstrates that a combination of Endo-ULBD, PKP, and PCD, performed through a single-channel, single-incision approach, may offer a promising, minimally invasive solution for elderly patients with coexisting OVCF and LSS. It offers an alternative to conventional fusion surgery, particularly for patients deemed to be high-risk surgical candidates. In the context of the extant literature, this technique represents a pragmatic, patient-specific strategy that strikes a balance between clinical efficacy and surgical safety. Further investigation in the form of prospective, multicenter trials is warranted.

## Data Availability

The raw data supporting the conclusions of this article will be made available by the authors, without undue reservation.
